# Pedro Pons’ sign

**DOI:** 10.11604/pamj.2014.17.177.4003

**Published:** 2014-03-07

**Authors:** Brahim Eljebbouri, Abad Cherif El Asri

**Affiliations:** 1 Department of Neurosurgery, Mohamed V Military Teaching Hospital, Rabat, Morocco

**Keywords:** Pedro Pons, low back pain, epiduritis, spine

## Image in medicine

64-year-old man farmar was admitted to our department with low back pain radiating to the lower extremities. He had a history of febrile illness and loss of appetite 2 years before admission. Four months after the start of the febrile illness, the patient started to complain of backache and spinal movements were restricted and painful. He had no other neurologic symptoms. Radiography of the spine revealed parrot's peak appearance (Pedro Pons’ sign) at the anterior superior end of the L3 vertebra which is characterized by osteosclerosis and osteophyte (A, arrows). Magnetic resonance imaging (MRI) after the administration of gadolinium revealed L2-L3 epiduritis, an erosive change of the anterior portion of L4 and L5, abnormal signal change with enhancement in T12, L2, L3, L5 and S1 body with probable spondylodiscitis in L2/L3 and L5/S1 levels (B). Pathological analysis of a specimen obtained with CT-guided aspiration revealed non caseating granulomas, which were culture positive for Micrococcus melitensis. Rose Bengal test for brucellosis was positive and Brucella standard tube agglutination confirmed this finding with 1/321 titer. Antibiotic treatment (doxycyline 200 mg/d and rifampicin 600 mg/d) was initiated and the patient's complaints were diminished gradually. He was discharged from hospital and recommended to complete his treatment to 90 days and a follow-up MRI showed sclerosis and reactive osteophyte formation indicating the healing phase of inflammatory tissue at the same levels. Pedro Pons’ sign, known as anterior superior end erosion, which occurs together with rounding of the vertebral end and level deformity, is a characteristic radiologic finding of brucellar spondylitis.

**Figure 1:**
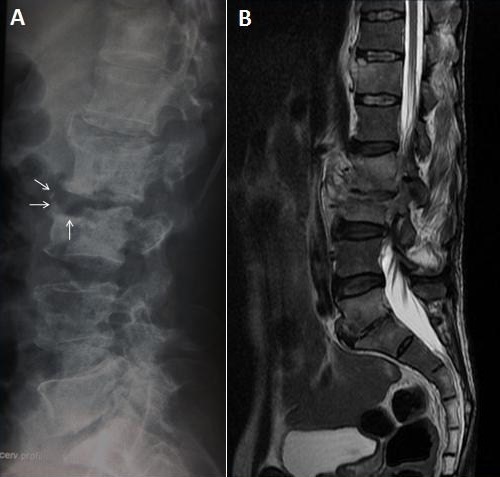
Radiography of the spine revealed parrot's peak appearance (Pedro Pons’ sign) at the anterior superior end of the L3 vertebra which is characterized by osteosclerosis and osteophyte (A, arrows). Magnetic resonance imaging (MRI) after the administration of gadolinium revealed L2-L3 epiduritis, an erosive change of the anterior portion of L4 and L5, abnormal signal change with enhancement in T12, L2, L3, L5 and S1 body with probable spondylodiscitis in L2/L3 and L5/S1 levels (B).

